# Causes of stress and conflict in the veterinary professional workplace – a perspective from Poland

**DOI:** 10.1186/s13620-020-00177-9

**Published:** 2020-11-17

**Authors:** Joanna Wojtacka, Wojciech Grudzień, Beata Wysok, Józef Szarek

**Affiliations:** 1 Department of Veterinary Public Health, Faculty of Veterinary Medicine, University of Warmia and Mazury in Olsztyn, Oczapowskiego St. 14, 10-718 Olsztyn, Poland; 2 Department of Pathophysiology, Forensic Veterinary Medicine and Administration, Faculty of Veterinary Medicine, University of Warmia and Mazury in Olsztyn, Oczapowskiego St. 13, 10-718 Olsztyn, Poland

**Keywords:** Veterinary profession, Animal owner, Conflict, Conflict-causing factors

## Abstract

**Background:**

The problems of burnout and the moral and ethical distress resulting from various kinds of conflict have been raised in the veterinary profession. However, their sources and inter-relationships have not been thoroughly recognized mainly due to the multidimensional nature of human interactions related to animal breeding, farming, welfare, prophylaxis and therapy. For the first time in Poland, an analysis of conflict and conflict-causing factors in veterinary practice has been conducted with the participation of veterinarians of various specialties and the owners of different animal species.

**Results:**

Conflict in the course of work is most often experienced by young veterinarians. The problems associated with communication between veterinarians and animal owners and unforeseen random situations are the general causes of conflict. Approved Veterinarians were identified by animal owners as the most common professional group associated with the conflict experienced .

**Conclusions:**

There is a lack of professional preparation by veterinary surgeons to cope with unpredicted stressful situations at work, resulting from an absence of appropriate educational input in this area. The animal owners do not understand the role and duties of Approved Veterinarians.

## Background

According to the Strategy of the Federation of Veterinarians of Europe [9] for the years 2015–2020, veterinarians have had a key role in many high profile areas, including animal health, animal welfare, food safety, environmental protection, the sustainable keeping of animals and antimicrobial resistance. As experts on animals and their needs, they play a crucial role in nearly every aspect of human-animal interaction [33]. According to data obtained from Polish National Veterinary Chamber, the number of active practicing veterinarians in Poland in 2017 was around 17,600, which makes it one of the largest groups of veterinarians in The European Union after Italy, Germany, Spain and France [8]. Over 4700 veterinarians in Poland work with farm animals, 7500 work with companion animals, 3200 veterinarians are employed full-time in permanent positions in Veterinary Inspection which means working in Chief, Provincial, District and Border Veterinary Offices based on a vertical chain of command. They are called Official Veterinarians. Veterinary Inspection also relies on the work performed by Approved Veterinarians. They are self-employed private veterinarians that are contracted by the Provincial Veterinary Officers to perform particular official activities, e.g. meat inspection; supervision of processing of food of animal origin, animal welfare monitoring at the level of production, transport and slaughter; carrying out official controls in the framework of combating infectious diseases in animals, etc. within a specified period of time. Their actions and decisions have legislative authority. There are also 1500 veterinarians working in higher education, 550 in pharmaceutical companies, including representatives working in the field of marketing and sale promotion, and 110 in the Polish army. With regards to the number of veterinarians per 1000 population, Poland is ranked similarly to France, Sweden and Croatia at the level of 0.2–0.27. This is half as much as in Ireland, Denmark, Norway, Luxemburg, Portugal Slovakia and Spain where the number of veterinarians per 1000 population ranges from 0.4 to 0.48. Survey data published by FVE [8] shows that in The European Union, there are about 233,300 veterinarians, of which approximately 186,000 are considered active. The number of companion animals in Europe is estimated to be approximately 157 million. However, there are also 59 million exotic animals, 417 million poultry, 360 million farm animals (cattle, pigs, sheep and goats), and 6 million horses. Despite decreasing trends in livestock numbers in recent years, the above description provides a basic evidence that there are two numerically unequal groups, veterinarians and animal owners who ostensibly share the same goals of animal health and welfare. Unfortunately, both parties do not often share a vision about how these goals can be achieved. The veterinarian’s responsibilities to animal owners and clients may be tempered by those to the patient, i.e. when there is a need to report an animal owner to relevant authorities [40]. Finally, conflicts in private veterinary practice mainly result from the inability to reconcile the differences between the veterinarian and the owner of the animal. Therefore, most of the current debate and literature revolves around normative, i.e. focused on the protection of the animal patients, conflicts in veterinary clinics, especially conflicts between the interest of the animal patient and the interest of its owner [33]. The presence of an animal makes the conflict-related nature of the relationship between two independent parties more complicated. By assumption, the veterinarian views the animal as a patient and his or her education, experience and work are to fulfil professional duties of care towards the animal. The animal owner frequently displays a degree of anthropomorphic perception of the animal or, on the contrary, an objectification of its existence. These ambiguous attitudes influence the standards of animal welfare adopted by humans and some dichotomous attitudes permit the exploitation and welfare violations against animals [35], e.g. obese dogs and cats suffer due to the fact that they are overfed by their owners, foxes and chinchillas spend their lives in tiny cages because the market for fur coats exists and goose fattening is practiced to meet the demands of the connoisseurs of *foie gras* all over the world. Nonetheless, the animal owner is not the only source of challenge that occurs in veterinary practice. A conflict may arise, e.g. as a result of a defect in feed formula [11, 30, 38] which leads to the deterioration of animal health and increased mortality or animal defects [2, 18] and makes an animal defective as a subject of the contract of sale. There are also feed producers or animal suppliers who become a party to the conflict. Veterinarians also face conflicts with different non-governmental organizations that fight for animal rights and draw public attention to animal welfare [36]. These kinds of conflicts are very challenging as they extend the sphere of the professional involvement of the veterinarian and reach an ethical and moral dimension leading not only to conflict itself but real distress [25, 29]. In addition, ignorance of the specifics of the work of veterinarians of various specialties often becomes a cause of friction. The Official Veterinarians constitute a special group which is decreasing in numbers each year according to FVE [9]. Veterinarians have been caught up in debates over issues such as genetically modified organisms, the emergence of such diseases as bovine spongiform encephalopathy (BSE), avian influenza, severe acute respiratory syndrome (SARS), the increasing frequency and destructiveness of extreme weather events and the loss of wild species that are important both ecologically and as food sources [32]. Generally, conflict and conflict-causing factors which pertain to medical doctors, residents, support staff or even students have been discussed in numerous publications [15, 16, 19, 27, 34]. In contrast, data concerning these problems in relation to veterinarians have not been widely recognized and focus more on moral distress and ethical context than the conflict itself. Few reports on the conflicts in veterinary healthcare teams show the extremely complex nature of this problem. The manifestation of negative attitudes including conflicting demands or even ignoring conflicts have a negative impact on veterinary team functions. Toxic environment is also indicated as a factor that leads to relationship and task conflicts in veterinary practice [24]. However, in this area, more research attention needs to be focused, especially on the functioning of the veterinary team.

The aim of the study was to examine and analyse the causes of the conflicts in the veterinary profession from the perspective of both the veterinarian and the animal owner.

## Methods

### Questionnaire survey

In 2017–2018, a survey of two different groups was conducted. One group consisted of veterinarians of various specialties who were practicing in Poland and were registered by the Polish National Veterinary Chamber. The other group consisted of animal owners of different species. The respondents completed the survey anonymously and voluntarily during different educational meetings. A total of 500 surveys were distributed among the analysed groups, 250 surveys among veterinarians, and 250 surveys among animal owners.

The questionnaire for veterinarians consisted of 13 questions. The first three questions served as supporting information concerning the general characteristics of the respondent, i.e. the type of work performed, gender, age group, and the remaining questions concerned various aspects of the conflict that the respondents faced in their professional work. Although they were close-ended questions, the” other” field gave the possibility to include one’s own statements in some cases. In some of the questions, respondents could select more than one answer.

The questionnaire for the animal owners consisted of five close-ended questions with the “other” field as described above. For one question, respondents could select more than one answer.

At the stage of survey distribution, no attempt was made to specifically target either veterinarians or animal owners because the study was designed to use an opportunistic sampling configuration [21].

### Statistical analysis

The statistical analysis of the data collected from the veterinarians and animal owners was performed in contingency tables, using Fisher’s exact test. Statistical significance was defined as *P* < 0.05.

## Results

### The survey of veterinarians

The data are shown in Table 1. Two hundred and twelve individuals responded to the survey (84.8% response rate). The respondents came from all over Poland. The majority of the veterinarians surveyed were employees in private practice employed on the basis of a contract of employment or another form (31.4%) and the owners of veterinary practices (28.6%). The others were Official and Approved Veterinarians (10%) and sales representatives working for pharmaceutical companies (11.4%). The vast majority of respondents were male (64.2%) while women accounted for 35.8%. The age of the most numerous group of the respondents ranged from 25 to 35 (37.7%). They were divided in terms of the frequency of conflicts occurring in their workplace with 22.6% of respondents declaring a conflict once a month, while a conflict occurring once a week, once a quarter, and less frequently than once a year was declared by 17% respectively for each response. The answer “once in six months” was indicated by 15.1% of veterinarians and “once every two weeks” and “once a year” by 5.6% of veterinarians for each answer.

**Table 1:** Survey responses given by veterinarians

Questions and answers	n (answers received)	Percent
**Q1**^a^**. What is the type of your employment?**		
Owner of veterinary practice	80	28.6%
Employee in veterinary practice	88	31.4%
Approved Veterinarian (self-employed, nominated by District Veterinary Officer for official activities, e.g. meat inspection)	28	10%
Official Veterinarian (employed full-time in Veterinary Inspectorate)	20	7.1%
Employee in a veterinary laboratory	4	1.4%
Representative of a veterinary pharmaceutical company working for marketing and sale promotion	32	11.4%
Employee in veterinary drugs warehouse	0	0%
Employee in a plant producing feed for poultry, swine or cattle	12	4.3%
Employee in the company involved in animal sale and purchase	8	2.9%
Other	8	2.9%
**Q2. What is your gender?**		
Female	76	35.8%
Male	136	64.2%
**Q3. Select your age.**		
25–35	80	37.7%
36–45	44	20.8%
46–55	48	22.6%
56–65	40	18.9%
Over 65	0	0%
**Q4. How often do you encounter conflicts at work?**		
At least once a week	36	17%
Once every two weeks	12	5.7%
Once a month	48	22.6%
Once a quarter	36	17%
Once in six months	32	15.1%
Once a year	12	5.7%
Less than once a year	36	17%
**Q5.**^a^ **Which animal groups do conflicts most often involve?**		
Exotic animals	4	1.5%
Wild animals	0	0%
Companion animals	76	29.2%
Ruminants	24	9.2%
Horses	0	0%
Swine	100	38.5%
Fur animals	0	0%
Poultry	56	21.5%
**Q6.**^a^ **With whom does the conflict arise?**		
Animal owner	132	43.4%
Employer	32	10.5%
Workmate	48	15.8%
Animal supplier	0	0%
Feed supplier	36	11.8%
Laboratory	4	1.3%
Official Veterinarian	36	11.8%
Other	16	5.3%
**Q7. How do you assess your work in terms of conflict-causing factors?**		
Low conflict	72	34%
Moderate conflict	100	47.1%
High conflict	40	18.9%
**Q8. How do you assess your work in terms of the number of conflicts when compared to other professions?**		
Less conflicting	10	4.7%
Equally conflicting	124	58.5%
More conflicting	60	28.3%
Definitely more conflicting	18	8.5%
**Q9. Do you often think about your conflict from work in your private time?**		
Practically always	88	41.5%
Occasionally	60	28.3%
Rarely	60	28.3%
Not at all	4	1.9%
**Q10. Does the conflict party often questions your authority?**		
It has never happened to me	28	13.2%
Rarely	64	30.2%
Occasionally	68	32.1%
Often	44	20.7%
Always	8	3.8%
**Q11. Do you think that conflicts in your work negatively affect you and your attitude towards other people?**		
No	28	13.2%
Rather no	72	34%
Rather yes	70	33%
Yes	28	13%
I have no opinion	14	6.6%
**Q12.**^a^ **What is the most important source of conflicts at your work?**		
Too high expectations of animal owners	92	29.9%
Failure in feed quality by its supplier	28	9.1%
Failure in quality by animal supplier	44	14.3%
Random situations	96	31.2%
Other	48	15.6%
**Q13.**^a^ **What is the most common cause of conflict escalation?**		
One party wants to show its superiority at all costs (“mine on top”)	72	23.1%
Money	88	28.2%
Cultural, religious and social differences	4	1.3%
Emotional attachment of the owner to the animal	36	11.5%
Lack of information and wrong conclusions	96	30.8%
Own internal conflict over the situation	4	1.3%
Other	12	3.8%

^a^ Asterisks indicate questions to which more than one answer was possible

Swine were identified as the group of animals that were most often associated with conflict (38.5%). They were followed by small animals (29.2%), poultry (21.5%), ruminants (9.2%), and exotic animals (1.5%) while conflicts most often concerned the owners of the animals (43.4%), workmates (15.8%) as well as feed suppliers and Official Veterinarians (11.8% each), employers (10.5%) and laboratories (1.3%).

Most of the veterinarians assessed their work as moderately conflict-related (47.1%) and low conflict-related (34%). They stated that the veterinary profession was equally conflict-related when compared to other professions (58.5%) although 28.3% of veterinarians responded that in their opinion more conflict was involved.

Despite the fact that animal owners undermined the authority of the veterinarian occasionally (32.1%) and rarely (30.2%) the majority of veterinarians (41,5%) almost always dwelled upon conflict experienced at work during their private time. Undermining authority happened often in 20.7% of cases, always in 3.8% of cases and 13.2% of veterinarians had never experienced such an attitude. One-third of respondents believed that conflicts rather negatively affected their attitude towards other people (33%). Another one-third (34%) thought in the opposite way. Still, “yes” or “no” answers were given by 13.2% of respondents in both cases.

The veterinarians indicated random situations (31.2%) and too high expectations of animal owners (29.9%) as the main causes of conflicts in their work. Feed quality defects or animal defects e.g. cribbing in horses were pointed out by 9.1 and 14.3% of respondents, respectively.

The respondents indicated that the conflict escalated most often due to: lack of information and wrong conclusions (30.8%), economic reasons (28.2%) and the willingness of one party to show its superiority at all costs (23.1%). Cultural, religious and social differences as well as one’s own internal conflict over the situation played minor roles in the process of escalation and account for 1.3% for both.

### The survey of animal owners

The data are shown in Table 2. Two hundred individuals responded to the survey (80% response rate). The respondents came from three voivodeships in Poland.

**Table 2:** Survey responses given by animal owners

	n (answers received)	Percent
**Q1. What is the main reason for conflicts between the animal owner and the veterinarian?**		
The veterinarian charge excessively for his/her services	20	10%
The veterinarian doesn’t want to conduct additional tests	36	18%
There are no treatment effects	60	30%
The animals must be treated frequently	0	0%
Feed producers attribute feed defects to mistakes in veterinary procedures and undermine the veterinarian’s authority	48	24%
Animal suppliers attribute animal defects (e.g. cribbing in horses) to mistakes in veterinary procedures and undermine the veterinarian’s authority	0	0%
Veterinarians do not want to work during the weekends	0	0%
Veterinarians talk about clients with others	0	0%
Veterinarians show their superiority	32	16%
Veterinarians criticize animal owners	0	0%
Veterinarians are incompetent	0	0%
Veterinarians are rude and arrogant	0	0%
Other	4	2%
**Q2. How do you assess the work of veterinarian in terms of conflict-causing factors?**		
Low conflict	156	78%
Moderate conflict	44	22%
High conflict	0	0%
**Q3. Which veterinarian do you most often have a conflict with?**		
Official Veterinarian (employed full-time in Veterinary Inspectorate)	6	3%
Approved Veterinarian (self-employed, nominated by District Veterinary Officer for official activities, e.g. meat inspection)	128	64%
Independently practicing veterinarian	16	8%
A veterinarian employed by feed manufacturer or animal supplier	50	25%
**Q4. How often do you encounter conflicts with veterinarian?**		
At least once a week	0	0%
Once every two weeks	0	0%
Once a month	0	0%
Once a quarter	0	0%
Once every 6 months	48	24%
Once a year	44	22%
Less than once a year	104	52%
I have not had such a conflict so far	4	2%
**Q5.**^a^ **What animal groups do conflicts most often involve?**		
Exotic animals	0	0%
Wild animals	0	0%
Companion animals	20	9.5%
Ruminants	14	6.7%
Horses	0	0%
Swine	60	28.6%
Fur animals	0	0%
Poultry	116	55.2%

^a^ Asterisks indicate questions to which more than one answer was possible

When asked about the main reasons for their conflicts with veterinarians, animal owners reported perceived poor response to treatment (30%); the fact that feed producers transferred feed quality defects to mistakes in veterinary procedures and undermining the veterinarian’s authority (24%); the fact that the veterinarian did not want to carry out additional tests (18%); the veterinarian showed his or her superiority (16%) and charged excessively for his/her services (10%).

The vast majority of responding animal owners believed that the work of a veterinarian was low conflict-related (78%) and only 22% stated it was moderately conflict-related. None of the animal owners replied that it was high conflict work.

When asked about the most common specialty of veterinarian they had had a conflict with, 64% of animal owners reported an Approved Veterinarian. This was followed by a veterinarian employed by a feed producer or animal supplier (25%), an independently practicing veterinarian (8%) and Official Veterinarian (3%). The conflict itself, according to the animal owners, usually occurred less than once a year (52%), once every six months (24%) or once a year (22%). It mainly concerned poultry (55.2%), swine (28.6%), and only involved companion animals (9.5%) and ruminants (6.7%) to a small extent.

**Fig. 1:**
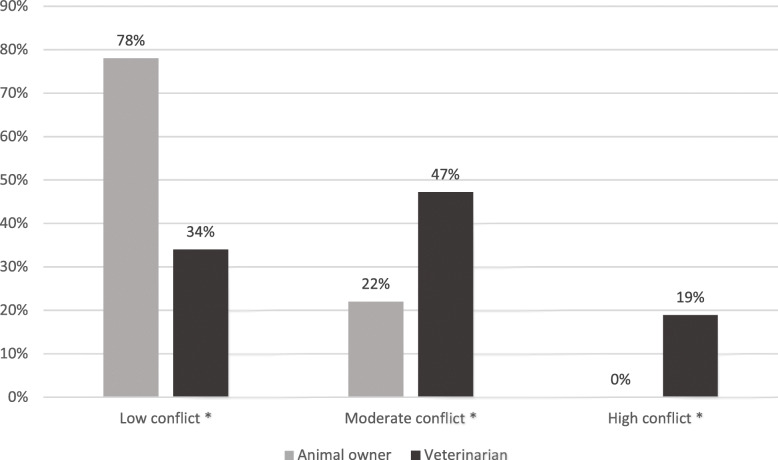
Percentage share of the answers given by veterinarians and animal owners to the question: “How do you assess the work of veterinarians in terms of conflict-causing factors?”. Asterisks indicate significant differences (P < 0.05)

**Fig. 2:**
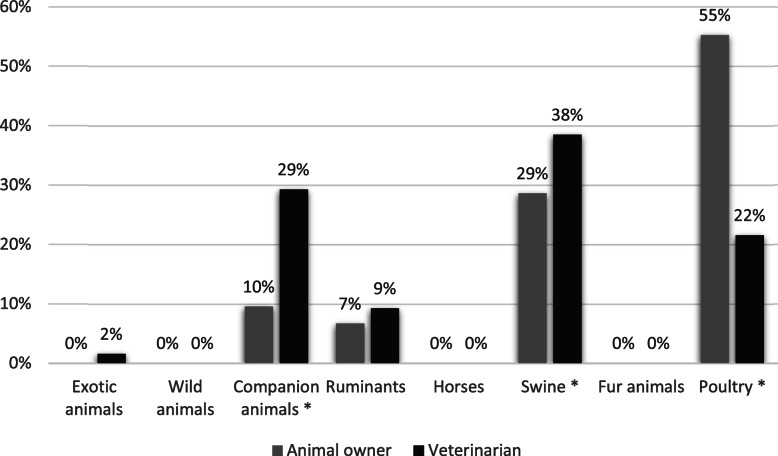
Percentage share of the answers given by veterinarians and animal owners to the question: “What animal groups do conflict most often involve?”. Asterisks indicate significant differences (*P* < 0.05).

In relation to two similar questions asked to both the veterinarians and animal owners statistically significant differences (*P* < 0.05) were found in terms of the conflict-causing factors in the veterinarian’s work (Fig. 1) and the reasons of conflict in terms of the groups of animals that conflicts most often involve (Fig. 2).

## Discussion

Since the 1950s, veterinary work has expanded from being concerned with the health and diseases of individual animals, to groups of animals (herd and flock health), human health (veterinary public health), business economics (which grew out of herd health), and, since at least the 1990s, the ecosystems that are the context for the health and wellbeing of all animals and humans (ecosystem health), [32]. The congested and lengthy curriculum of undergraduate studies in veterinary medicine in Poland has been focused on theoretical and practical approaches to prepare new veterinarians to enter the market. It has been developed by different governing bodies that assume that the ‘product’ of a vocational degree course such as Veterinary Medicine should be a competent professional equipped for their first day in practice [17]. Nevertheless, more attention has recently been paid in Poland to lifelong learning of veterinarians and the voluntary score collection program which was implemented by the National Veterinary Chamber to encourage veterinarians to upgrade their knowledge and skills systematically.

According to data published by FVE [8], the veterinary profession is practiced by relatively young veterinarians among whom 44% are aged under 40. This is in line with the profile of respondents surveyed in the current study and recent research conducted in Poland on the group of Veterinary Food Inspectors [37] and indicates that the conflict in professional work is experienced mainly by veterinarians at the beginning of their carrier path and employed full time in a veterinary practice owned by another person. In the opinion of veterinarians, a conflict situation occurred in their workplace on average once a month. In the opinion of the animal owners, conflict with a veterinarian occurred less than once a year, which may be due to the fact that apart from single pet owners, animals are generally the means to implement business plans, and emotional involvement in breeding and farming is not the main reason for contacting a veterinarian to ask for his/her services [10]. The animal owners tend to forget that veterinarians fundamentally do not have the same responsibility to care for clients as for their patients [40]. Moreover, animal owners pointed to poultry as the main source of conflict with the veterinarian and, in their opinion, the perceived lack of treatment effect was the most common cause of conflict. At this point, attention should be drawn to the statistically significant differences in the perception of animal groups that caused the highest number of conflicts according to veterinarians and animal owners questioned in the survey. First, both parties indicated different animal species, i.e. swine and poultry, respectively. According to Babińska et al. [1] conflicts related to poultry are, in fact, the most common subjects of ligation. The above suggests that conflicts related to swine, despite the fact that they occur relatively often, can be resolved at the level of mediation between both parties and are rarely finalized before a court. Both, poultry- and swine-based conflicts between the animal owner and the veterinarian, as they concern large-scale production, are in principle entangled by other parties. In this context, the respondents representing veterinarians and owners reported employees of feed suppliers, sales representatives and animal suppliers as sources of conflict. However, it should be noted that animal owners experienced such conflicts twice as often as veterinarians who assessed their work as moderately conflict-related, although no more conflict-related than in other professions. The animal owners reported that the majority of conflict situations arised in relations with an Approved Veterinarian who performed duties appointed ex officio. In Poland in 2016 and 2017, there were more than 6000 veterinarians serving as Approved Veterinarians, of which more than a half were nominated for more than one activity. The Official Services protect human health through the prevention of zoonoses and the hygienic control of foodstuffs, and by helping to improve primary and secondary zootechnical production and, thus the socioeconomic welfare of the population [22]. However, such a wide approach may be misunderstood by the animal owners for many reasons, i.e. economic, social and cultural constraints and perceptions that may present obstacles to the establishment of a hygiene culture and a commitment to veterinary public health (VPH), [39].

The results of the survey indicate that veterinarians almost always transferred the conflict to their private sphere of life. Surveys conducted in the United Kingdom in 2014 have shown that over 90% of veterinarians consider their work to be stressful [6]. Numerous studies in the field of work and family confirm the side effects of stress resulting from conflict situations, as also affecting colleagues, spouses, children and the entire community with whom a person affected by professional stress comes into contact with [28]. Nevertheless, when analysing the results of the survey of Polish veterinarians, it was also noted that conflict situations in their work had not affected the perception of other people they had met in their professional life, and authority had rarely been questioned. This shows why, from the point of view of animal owners, the work of a veterinarian was not very conflict-related. However, some answers received from veterinarians may indicate the predisposition of the respondents to the occurrence of phenomena correlated with stress. These were contradictory answers given to the questions regarding the frequency of conflicts in professional life, the fact of analysing the conflict in their private time and declaring the lack of influence of these two factors on the perception of other people. Studies conducted in Australia have shown that veterinarians described higher levels of depression, anxiety, stress and burnout than the general population [13]. Bartram et al. [4] found unexpected outcomes and possible client complaints and litigation were among the greatest contributors to work-related stress in veterinary surgeons in the UK. Polish veterinarians declared their work was not very conflict-related but admitted to transfer conflicts from their worplace to the sphere of private life. Such conditions may predispose veterinarians to the burnout syndrome that was described by veterinarians from Australia, New Zealand, USA, UK and Belgium [26]. Moreover, as indicated by studies conducted among veterinarians in Belgium, the specialty had no effect on the frequency of burnout. It occurs at a comparable level among veterinarians treating companion animals, farm animals and in the so-called mixed practices [12]. The respondents-veterinarians in the current study reported that random situations, the excessively high expectations of animal owners, the escalation of the conflict caused by the lack of information, and drawing incorrect conclusions were the main sources of conflicts. The economic aspect was also highlighted and it is in accordance with what has been repeatedly raised in the literature in regard to bearing the costs associated with veterinary care as a barrier between a veterinarian and the animal owner [7]. Moreover, apart from the financial and ethical dimensions of the conflict, there is also a conflict of interest that can be different for the animal and the client who pays for the treatment [5, 14, 31, 32]. Undeniably, managing people and emotions in such complex circumstances requires practice, time and patience all at once [20]. Actions to resolve the conflicts peacefully are necessary, not only to satisfy the conflicting parties but also to prospectively avoid mental health disorders and related consequences.

The multidimensional impact of problems that become a source of conflict in the work of veterinarians is confirmed by the fact that in 2015 the vast majority of veterinarians in Europe, including Poland, indicated that Veterinary Schools did not equip graduates with sufficient skills [8]. Moreover, the veterinary undergraduate course has been described as having the potential to stifle communication skills and emotional intelligence [3], which are the main factors in stress management and conflict handling [23]. The curriculum in Poland does not include subjects devoted to stress or time management, approaches to difficult clients or social techniques useful in conflict situations at work. The students can follow the subjects related to management of veterinary practice or rules of the labor market but most of them function as elective subjects receiving little interest. Fundamental changes in the teaching program cannot be implemented ad hoc and need thorough planning. In addition, further research is required and more data must be collected to characterize and authenticate the root cause of the issue, i.e. the lack of training of the graduate. Thus, if the Veterinary School does not provide a curriculum corresponding to current demands, then there is a role for organizations that should provide resources such as lifelong learning and continuing professional development workshops in verbal and non-verbal communication and the maintenance of mental, spiritual and emotional balance of veterinarians [20].

## Conclusions

This study provides data to support the move towards the inclusion of professional skills attributes including communication skills and coping the mechanisms to deal with stressful situations in the workplace in the undergraduate curricula of veterinary medicine in Poland. Further research in defining and implementing day one competences for veterinary graduates is required.

Animal owners show deficiencies in understanding the role, tasks and responsibilities of veterinary surgeons fulfilling official duties within the activities assigned to veterinary public health.

## Data Availability

The datasets generated and/or analysed during the current study are available from the corresponding author on request.
